# Outcomes of three different ways to train medical students as ultrasound tutors

**DOI:** 10.1186/s12909-019-1556-4

**Published:** 2019-05-02

**Authors:** Nora Celebi, Jan Griewatz, Nisar Peter Malek, Tatjana Hoffmann, Carina Walter, Reinhold Muller, Reimer Riessen, Jan Pauluschke-Fröhlich, Ines Debove, Stephan Zipfel, Eckhart Fröhlich

**Affiliations:** 1 PHV dialysis center Waiblingen, Beinsteiner Straße 8/3, 71334 Waiblingen, Germany; 2 Competence Centre for University Teaching in Medicine, Baden-Wuerttemberg, Elfriede-Aulhorn-Str. 10, D-72076 Tuebingen, Germany; 3 Department of Internal Medicine I (Gastroenterology, Hepatology, Infectious Diseases), University Hospital Tübingen, Otfried-Müller-Str. 10, 72076 Tübingen, Germany; 4 Eberhard-Karls University, Geissweg 5/3, 72076 Tübingen, Germany; 5 James Cook University, 1/14-88 McGregor Rd, Smithfield, QLD 4878 Australia; 6 Department of Internal Medicine VIII (Intensive Care Unit), University Hospital Tübingen, Otfried-Müller-Str. 10, 72076 Tübingen, Germany; 7 Department of Womens Health, University Hospital Tübingen, Calwerstraße 7, 72076 Tübingen, Germany; 8 Department of Neurology, Inselspital, Bern University Hospital, University of Bern, Freiburgstrasse 18, 3010 Bern, CH Switzerland; 9 Department of Internal Medicine VI (Psychosomatic Medicine), University Hospital Tübingen, Osianderstr. 5, 72076 Tübingen, Germany

**Keywords:** Undergraduate medical education, Ultrasound education, Student instructor education, Ultrasound tutor skill acquisition, Internship, Course

## Abstract

**Background:**

In order to provide faculty-wide undergraduate ultrasound training in times of scarce resources, many medical faculties employ trained peer-student tutors to oversee the hands-on training. However, data to guide the training of ultrasound peer-student tutors are scarce. We conducted a prospective quasi-randomized study to assess the gain in theoretical knowledge and practical scanning skills of peer-student tutors who were trained with a course only, an internship only, or the combination of a course and an internship.

**Methods:**

A total of 44 peer-student tutors were trained by a one-week course only (C-Group, *n* = 21), by an internship only (I-Group, *n* = 10) or by a course and an internship (CI-Group, *n* = 13). Prior to and after the completion of the training the peer-student tutors completed an MC-test (theoretical knowledge) and an OSCE (practical scanning skills).

**Results:**

With all three education concepts, the peer-student tutors had significant and comparable gains in theoretical knowledge (C-group + 90%, I-group + 61.5%, CI-group + 114.0%) and practical scanning skills (C-group + 112.0%, I-group + 155.0% and CI-group + 123.5%), all *p* < 0.001.

**Conclusion:**

Peer-student tutors, who were trained with a course or an internship or a course and internship improved their theoretical knowledge and their practical scanning skills significantly and to a comparable degree.

## Background

Ultrasound is one of the prime imaging techniques in clinical medicine, especially in emergency medicine [21]. Some basic techniques are comparably easy to learn and have a positive impact on patient outcome. Thus, there is a broad consensus to incorporate ultrasound into the undergraduate curriculum in order to ensure that novice physicians have these basic skills ([3, 6, 8, 10, 14, 16, 17, 19, 20, 23, 26, 28]).

In order to perform ultrasound examinations, the student has to master theoretical knowledge and acquire the ability to generate adequate images [3, 4]. For teaching the latter, most medical schools rely on hands-on sessions supervised by students trained in conducting musculoskeletal ultrasound, we refer to them in this article as “peer-student tutor” [6, 27].

While this concept has been proven to be effective and well accepted for various skills, data to guide the training of ultrasound peer-student tutors are scarce [7, 12, 15, 18, 27]. According to the framework by the AMEE regarding peer assisted learning, several questions should be addressed when implementing such a learning concept. Questions 7–9 deal with the recruitment and training of the peer-student tutors, specifically question 8 “what training will tutors require and how will this be provided?” and question 9 “how else will tutors prepare themselves and reflect afterwards” [25]. The authors suggest to implement didactical training as well as content specific training according to the skill addressed in the specific peer assisted learning-project [25].

Fox and colleagues found that a four week internship is superior to a two week internship in order to learn emergency ultrasound [11]. In a study by Ahn and colleagues, peer-student tutors who were trained for four weeks were rated higher by their tutees than peer-student tutors who were trained for two weeks [1]. For echocardiography even a three week internship was not enough to train the peer-student tutors to faculty staff level, and in the study by Ahn the echocardiographic planes were rated as difficult [1, 18]. In comparison, peer-student tutors who received only a 30 min training on musculoskeletal ultrasound followed by a one week self-directed learning phase were able to teach ultrasound skills with results equal to faculty members [15]. Thus, not only the mode and length of the internship but also the complexity of the skill must be taken into consideration; comparisons of teaching concepts for ultrasound across different skills are difficult to interpret.

In theory, there are three different concepts to provide direct supervised hands-on training to future ultrasound peer-student tutors: An internship in ultrasound laboratories, a course, or the combination of both. A course has the advantages of a high capacity to train a large number of peer-student tutors simultaneously and the full control over the content. However, the time students spent practicing the actual scanning is limited and the organizational effort to implement a course is high. An internship on the other hand is easy to implement and the future peer-student tutors have plenty opportunity to practice scanning and see pathologies. However, the number of students who can be trained is limited by the capacity of the ultrasound-laboratories and there is only limited control over the pathologies the future peer-student tutors are going to encounter.

Thus, the students trained by a course alone should have a practical disadvantage, the students trained by an internship alone a theoretical disadvantage. To offer a course followed by an internship should combine the strengths of both concepts.

Since most ultrasound curricula for undergraduate medical education focus on rather simple, easy to learn skills, it is an empirical question whether the above described differences between the teaching approaches are practically relevant and whether one concept of training future ultrasound peer-student tutors is superior to another [2, 6, 9, 14]. Thus, the results of our study will contribute to the success of future ultrasound peer-student tutors by assessing the gain in theoretical knowledge and practical scanning skills of each strategy, and, if the training strategies appear comparably effective, to justify the selection of the strategy that fits the individual teaching context best.

We therefore trained three groups of ultrasound peer-student tutors using the three different most often described approaches in the literature concerning ultrasound peer-student tutor education: a course only, an internship only, and the combination of both. The gains in theoretical knowledge and the technical ability to acquire and interpret ultrasound-images were measured in a pre-post design.

## Methods

### Study design

This prospective, quasi-randomized study used a pre-post design to assess the gain in theoretical ultrasound knowledge and practical scanning skills of medical students applying for tutorship under three different training regimes.

### Participants

The training was advertised on the faculty’s central message board and students from 3rd to 5th year were accepted in the order of their application for tutorship without further selection criteria. All peer-student tutor applicants gave written consent to participate in this study with the opportunity to withdraw at any time.

In the course only-group (C-Group), 23 students were accepted for the training and 21 finished, in the internship only-group (I-Group), 12 students were accepted and 10 finished the training. A total of 29 students were accepted for the course plus internship training (CI-Group), 13 finished the training.

### Training

For two semesters we concomitantly offered potential peer-student tutors a course only or an internship only training concept. In the following two semesters we offered a training that encompassed both, a course and an internship.

In the course only-training, faculty members delivered lectures on the various ultrasound topics followed by hands-on phases in which the peer-student tutor applicants examined each other supervised by faculty members and experiencedpeer-student tutors. The course took place over five whole days and was accompanied by a script covering all the topics and picture examples of pathologies.

The topics were: Physics, artefacts, handling of the ultrasound device, documentation, liver, gallbladder, bile ducts, retroperitoneal structures with vessels and lymph nodes, pancreas, spleen, peritoneal cavity, kidneys, bladder, uterus, systematic examination abdomen, FEEL (Focused Echocardiography in Emergency Life Support), throat, thyroid, jugular veins, carotid arteries, lymph nodes, basic color doppler, compression duplex sonography of the deep veins, thorax, lung, FAST, and E-FAST (Extended Focused Sonography in Trauma).

In the internship only-group the peer-student tutor applicants received the same script and interned for a minimum of 21 and a maximum of 35 days in four different ultrasound laboratories. The peer-student tutors were advised to try to cover as much content from the script as possible and to rotate though the laboratories whenever they felt that they had sufficiently mastered the examinations offered in the respective ultrasound laboratory.

In the course plus internship group the peer-student tutors sat the one week course described in the course only-group followed by a 21 day internship in which the students rotated through seven different ultrasound laboratories on a three-workday schedule. The students received the same script as the other students.

### Assessment

Prior to their training the students filled in a questionnaire for demographic data and sat a multiple choice test comprising ten questions on the theory of ultrasound. The topics of the test were not covered in the curriculum so far. In addition, the students demonstrated their scanning proficiency in an objective structured clinical examination (OSCE) in which they were asked to depict and label 15 anatomical structures in a maximum of three images within eight minutes. All OSCEs were performed on identical ultrasound machines (Accuson X 300, Siemens, Erlangen, Germany) on healthy peer students with optimal imaging conditions. Two faculty members sat the same OSCE in order to provide a benchmark. Directly after completion of their training, the peer-student tutors sat the identical assessments again.

### Rating of OSCEs

Two experienced faculty members independently rated the pictures. All images were mixed assigning random numbers to them so that the raters were blinded as to the origin of the pictures (student name and phase of the study). Additionally, potential observer drift was controlled for by one rater rating the OSCE pictures in ascending random number order, the other in descending order. For every label in which the correct anatomical structure could be clearly identified in the picture the raters awarded one point, if the structure corresponding to the label was potentially visible the raters awarded half a point. The overall OSCE rating was then achieved by adding up the achieved points and averaging the two independent ratings.

### Statistical analysis

A power analysis based on a prior study with the same assessment revealed that a group size of *n* = 10 per group was sufficient to achieve a power in the excess of 80% to find an improvement of 7 points in their OSCE scores as significant at an alpha level of 5%.

Inter-rater agreement was assessed by linear regression and an intraclass correlation coefficient (ICC) [22]. Categorical variables were displayed as percentages. Numerical variables proved to be reasonably normally distributed and therefore means (± standard deviations (SD)) were used for descriptive purposes and supplemented by 95% confidence intervals (95% CIs) for the main outcome measures. Paired t-tests were employed for pre/post statistical comparisons within groups; one-way ANOVA was used for tests between groups. For all tests, a *p*-value of less than 0.05 was regarded as statistically significant.

### Ethics

The local ethics committee waived the need to give consent (decision number 667/2016BO2). Thus, enrolment into the student tutor program was deemed as consent.

## Results

A total of 44 peer-student tutors completed the study. Of these, 10 were in the I-group, 21 in the C-group and 13 in the CI-group. The characteristics are shown in Table 1.

**Table 1:** Demographic Data and Group Comparisons at Baseline

	I-group (*n* = 10)	C-group (*n* = 21)	CI-group (*n* = 13)	*p*-value
Male Gender 13 m*	60.0% (6/10)	55.6% (5/9)	50.0% (6/12)	*p* = 0.40
Age (mean/stddev) 5 m*	24.1 (±2.3)	25.6 (±3.1)	25.4 (±2.8)	*p* = 0.41
Semester (mean/stddev) 3 m*	7.1 (±1.5)	8.6 (±1.1)	6.9 (±1.6)	*p* = 0.002
Previous Tutorship 4 m*	20.0% (2/10)	33.3% (6/18)	41.7% (5/12)	*p* = 0.57
Previous ultrasound experience 3 m*	30.0% (3/10)	50.0% (9/18)	38.5% (5/13)	*p* = 0.61
Previous participation in ultrasound course 3 m*	40.0% (4/10)	33.3% (6/18)	38.5% (5/13)	*p* = 0.98
Previous participation in didactics course 5 m*	20.0% (2/10)	50.0% (8/16)	30.8% (4/13)	*p* = 0.30
MC pre-Scores 1 m*	5.2 (±1.8)	5.0 (±1.9)	4.2 (±1.9)	p = 0.41
OSCE pre-Scores	3.3 (±1.5)	5.0 (±2.7)	3.4 (±1.6)	*p* = 0.047

* m = nr of missing information

The inter-observer-agreement between the two independent raters of the OSCE images was very high with an intra-class-correlation (ICC) of 0.97 (95% CI: 0.95–0.98).

The groups were essentially comparable, only the C-group revealed slightly better pre-OSCE results than the two other groups. There was a slight, nonsignificant trend towards an older age and more ultrasound exposure in the C-group compared to the other groups. Theoretical knowledge and practical ultrasound skills, pre- and post-training, as well as the observed gains (in absolute and in relative terms) within the three groups are displayed in Table 2.

**Table 2:** Theoretical Knowledge and Practical Skills Gains within Groups

	Pre	Post	Gain	Relative Gain	*p*-value
Scores MC Test Results (Theoretical Knowledge)					
I-group	5.2 (±1.8)	8.4 (±0.8)	3.2 (±1.8)	61.5%	*p* < 0.001
C-group	5.0 (±1.9)	9.5 (±0.5)	4.5 (±2.0)	90.0%	*p* < 0.001
CI-group	4.2 (±1.9)	9.0 (±0.7)	4.8 (±1.7)	114.0%	*p* < 0.001
OSCE Tests (Practical Skills)					
I-group	3.3 (±1.5)	8.4 (±2.1)	5.1 (±0.6)	155.0%	*p* < 0.001
C-group	5.0 (±2.7)	10.6 (±1.7)	5.6 (±2.7)	112.0%	*p* < 0.001
CI-group	3.4 (±1.6)	7.6 (±1.8)	4.2 (±1.6)	123.5%	*p* < 0.001

All teaching groups improved substantially, significantly, and comparably in both theoretical knowledge and practical skills (all *p* < 0.001, paired t-tests).

**Fig. 1:**
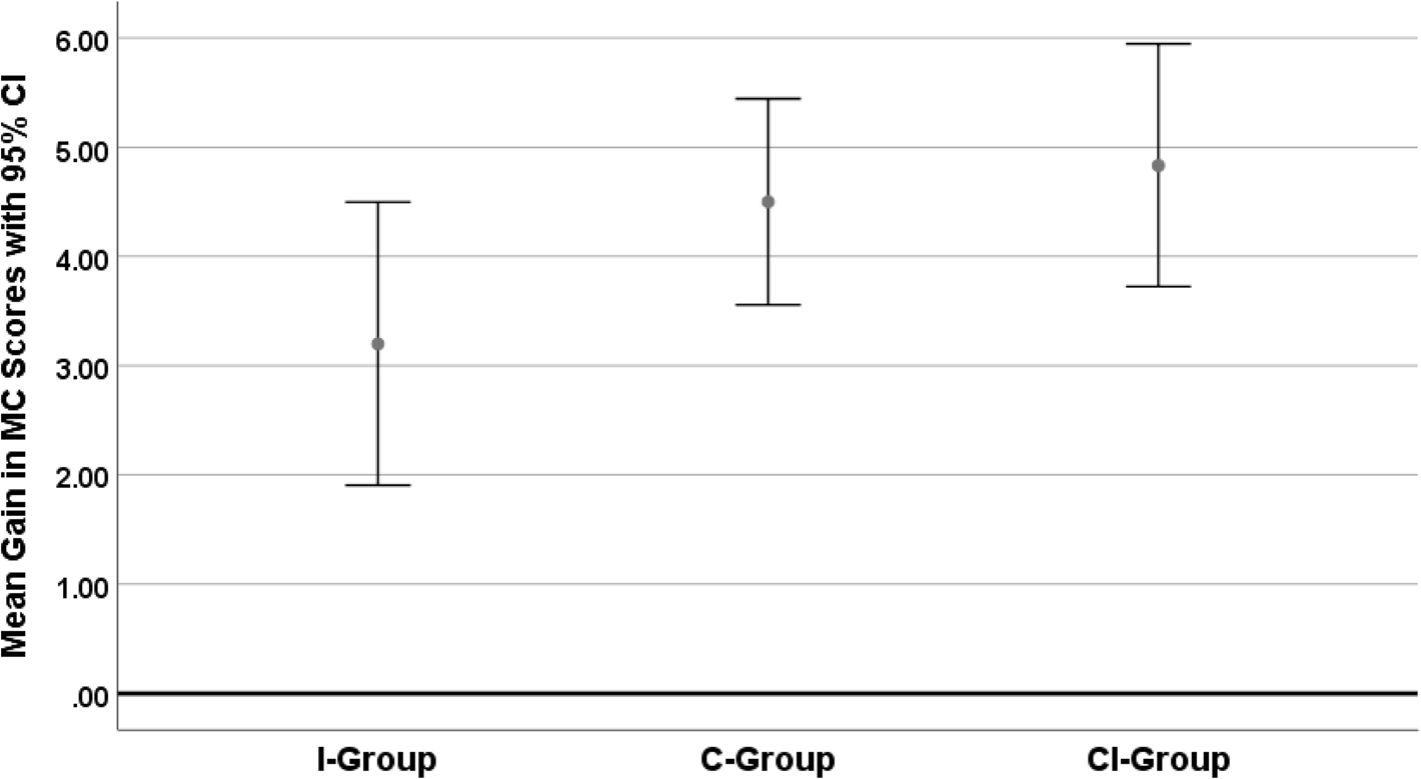
Mean gains in MC-Score (together with 95% confidence intervals) of students trained with an internship only (I-group), a course only (C-group), or a course and an internship (CI-group)

**Fig. 2:**
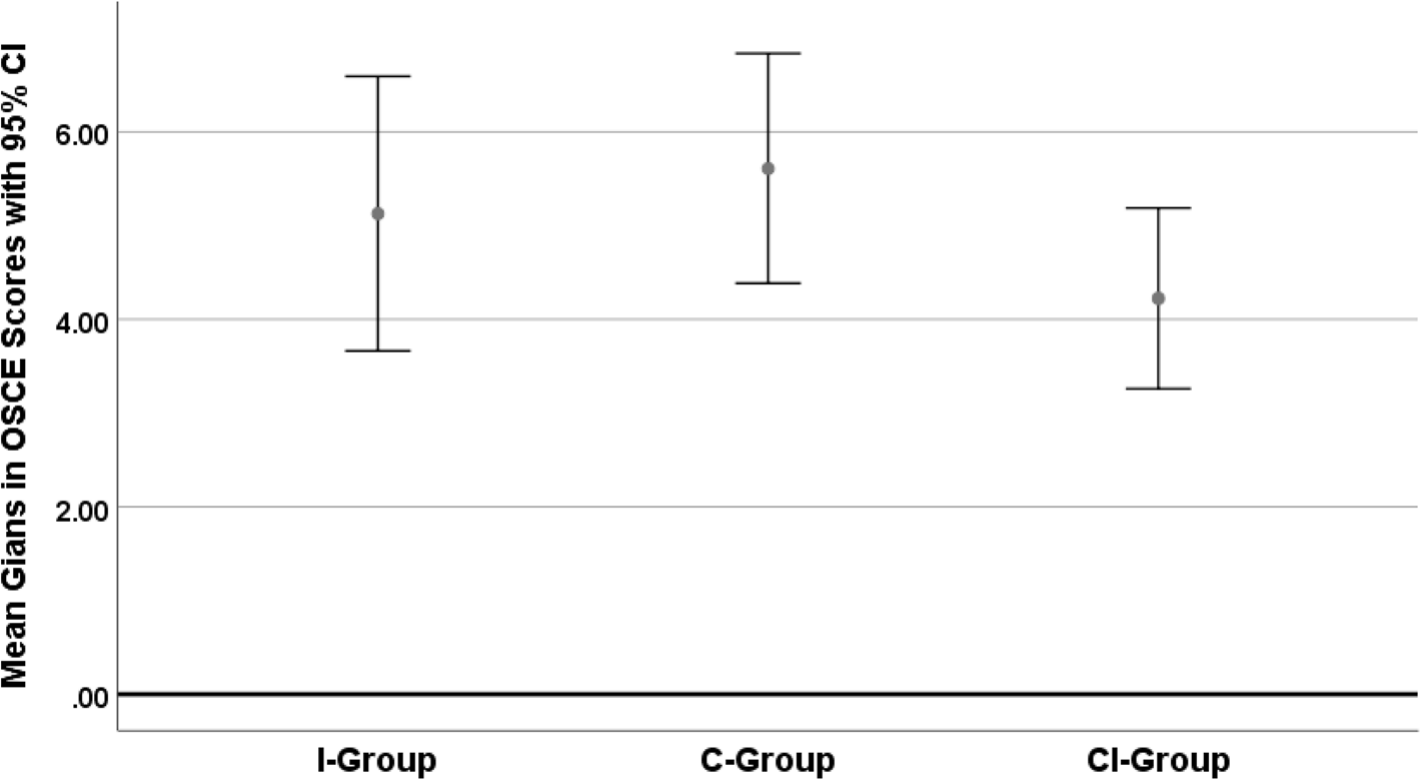
Mean gains in OSCE-Score (together with 95% confidence intervals) for students trained with an internship only (I-group), a course only (C-group), or a course and an internship (CI-group)

The main outcome measures (mean gains) are displayed together with their 95% confidence limits in Figs. 1 and 2.

## Discussion

Many medical schools which offer ultrasound education for undergraduate medical students employ a peer-teaching concept with trained ultrasound peer-student tutors [13, 27]. However, data guiding the education of these ultrasound peer-student tutors are scarce.

Our study provides empirical data for improvements in theoretical knowledge and practical scanning skills of ultrasound peer-student tutors for different teaching concepts for ultrasound peer-student tutors. We assessed the knowledge gain of ultrasound peer-student tutors who were trained by a course, an internship, or the combination of a course and an internship.

All three groups revealed substantial, significant and comparable gains with a slight tendency for the I-group to show a less pronounced theoretical improvement. However, most notably, the improvement in scanning skill in the C-group who was trained for one week only was comparable to the I- and CI-group who had several weeks to practice the scanning.

In the scarce literature the influence of the length of the training on the acquisition of scanning skills is variably discussed with results seemingly depend on the ultrasound skill in question. Based on the limited evidence available so far, Tarique et al. concluded in their review, that a longer training results in better student tutor performance [27]. In previous studies from our group, for abdominal ultrasound, a three week training was sufficient to bring student tutors to a level equal to that of faculty staff, while this was not the case for echocardiography skills [7, 18]. However, we did not assess whether an even shorter training for abdominal ultrasound or a longer training for echocardiography would have changed the results. Ahn and colleagues trained peer-student tutors for two and four weeks and found, that longer training resulted in higher rating by their tutees, but they taught a multitude of skills like abdominal ultrasound, ocular ultrasound, musculoskeletal ultrasound, echocardiography, lung ultrasound and vascular ultrasound [1]. For multiple emergency ultrasound skills, Fox and colleagues found that students after a four week internship outperformed their peers after a two week internship in the emergency department in an theoretical test [11].

In contrast, Knobe and colleagues trained peer-student tutors with a 30 min instruction on musculoskeletal ultrasound only, followed by a one week self-directed learning phase and found teaching results equal to faculty members [15]. Wakefield et al. recruited faculty members not performing ultrasound on a regular basis for their undergraduate curriculum, trained them with a half-day course and found this concept feasible, although they did not assess the gain in knowledge/ skill gain or the teaching success of these tutors [28].

Peer assisted learning concepts are employed for a vast scope of skills in medical schools, and in most concepts the peer-student tutors are educated with a combined didactical and content-specific approach [5]. Even for some practictal skills, very short training concepts are reported in the literature [5]. For most skills these concepts are equal to faculty teaching [24].

There are some limitations of our study.

The main aim was to assess whether the teaching methods for the tutors were able to generate significant gains in knowledge and skills. Thus, our study was only powered to detect a within-group improvement, not to detect differences between groups. A much larger study would have been needed and should be conducted in the future to explicitly assess whether one particular method is superior to another. According to our data, a group size of more than 160 student tutors per group would be necessary to answer this question. We already pooled data from four consecutive terms, so several universities would have to implement the same program in order to conduct such a study.

We did not randomize the groups and there were slightly higher scores in the pre-OSCE-assessment of the C-group, thus there is a possibility, that our study groups were not ideally comparable. In addition, in comparison to the two other groups, a higher proportion of the CI-group did not perform the post-assessment for unknown causes, for this reason we cannot exclude the potential of some selection bias in this CI-group.

The assessment of the practical skills was designed to provide a rather conservative reliable measurement to compare the ultrasound scanning skills of an individual prior and after the training. Since the students had a time and image-limit, the test is likely to underestimate the actual scanning ability.

## Conclusion

Ultrasound peer-student tutors improved substantially, significantly and comparably in both theoretical knowledge and practical scanning ability regardless whether they were trained by an internship only, a course only, or the combination of a course and an internship. Thus a medical faculty striving to implement an ultrasound curriculum using student tutors can choose a training method that best suits their specific circumstances.

### Practice points

- Ultrasound peer-student tutor training using an internship has the advantage of a low effort to implement a peer-student tutor training program and a lot of opportunity to practice clinical scanning skills. Disadvantages are a low capacity and a limited control over the content.
- Ultrasound peer-student tutor training using a course has the advantage of a high capacity and full control over the content. Disadvantages are a high organizational effort and little time to practice clinical scanning.
- The combination of course and internship combines the advantages and the disadvantages of both concepts.
- In our study, peer-student tutors trained with any strategy showed comparable theoretical and practical skill gain.
